# Comparing the accuracy of point-of-care with laboratory (capillary, venous, and arterial) blood glucose levels in critically ill patients with and without shock

**DOI:** 10.1186/s13104-022-06256-0

**Published:** 2022-12-17

**Authors:** Abdulaziz Alshaer, Basma A. Badgheish, Zahra Hashim Alsadah, Khalid Sewify, Sarah Alghazal, Sarah Alzahrani, Abeer Qadi, Reham Alqahtani, Ghadeer Abdullah Farsani, Amal Shilash

**Affiliations:** 1 King Fahd Military Medical Complex, Abqaia Road, Dhahran, 31932 Saudi Arabia; 2 Surgical Intensive Care Unit, King Fahd Military Medical Complex, Dhahran, Saudi Arabia; 3 Critical Care Unit, King Fahd Military Medical Complex, Dhahran, Saudi Arabia; 4 Laboratory Medicine, King Fahd Military Medical Complex, Dhahran, Saudi Arabia; 5 Intensive Care Unit, King Fahd Military Medical Complex, Dhahran, Saudi Arabia

**Keywords:** Blood glucose, Intensive care unit, Patients in shock, Laboratory

## Abstract

**Objectives:**

To compare the accuracy of point-of-care capillary and venous/arterial samples to laboratory testing of venous/arterial samples in critically sick shocked and non-shocked patients. This is a prospective case–control study including capillary, venous, and arterial blood samples from 268 critically ill patients. The King Fahd Military Medical Complex in Dhahran, Saudi Arabia, was the site of this investigation.

**Results:**

We were able to obtain data on 268 patients for this investigation. POCT and lab findings of venous and central blood did not differ significantly (P = 0.389 and 0.208), while POCT indicated somewhat higher results with venous glucose concentrations of 10.18 and 10.05 (POCT and lab tests respectively) and 9.18 and 9.54 (POCT and lab tests respectively). In addition, the mean differences between POC and laboratory analyses of venous, arterial, and central glucose were 0.13, − 1.75, and − 0.36 mmol/L for venous, arterial, and central glucose, respectively. Except for arterial blood glucose, we did not observe a significant difference between POCT and routine laboratory analysis of glucose concentrations in critically ill patients. Compared to laboratory blood analysis, the use of POCT is marginally accurate, with no difference between shocked and non-shocked patients.

## Introduction

Maintaining normoglycemia in patients with preexisting diabetes or stress-induced hyperglycemia is one of the most crucial components of intensive care. It has been determined that hyperglycemia has a deleterious effect on these patients [1, 2]. Appropriate management of hyperglycemia has been proven to have a considerable influence on lowering mortality and hospital length of stay, preventing acute renal injury, and facilitating a quicker weaning from mechanical breathing [1, 3, 4]. In addition, hypoglycemia is related with adverse effects [5] and has been identified as an independent predictor of mortality [6, 7] in a number of investigations.

Many hospitals utilize point-of-care (POC) glucose meters to monitor glycemic status in order to meet these aims. Patients requiring strict glycemic control, for whom waiting for central laboratory findings makes rapid modifications and management of glucose level in therapy difficult, benefit greatly from the mobility, simplicity of use, and immediate availability of results that POC glucose meters offer. In stable outpatients, the majority of glucometers were found to be reliable; however, among critically ill patients, a number of confounding factors, such as hematocrit, oxygenation, acid–base disturbance, temperature, and shock states, were reported to interfere with POC glucometers [10, 11].

Considering the presence of shock, several mechanisms have been proposed as possible explanations for its impact on the accuracy of POC glucometers, including peripheral vasoconstriction in hypoperfusion states, which could result in increased glucose extraction by tissues due to low capillary flow, leading to a falsely underestimated glucose measurement with capillary blood [12, 13]. Several papers on monitoring blood glucose levels in critically ill patients demonstrated significant variance between point-of-care and laboratory values, but did not distinguish between shocked and non-shocked patients [4, 5] [1, 14–19]. Previous studies conducted on critically sick patients contained a small number of measures taken from individuals in shock, resulting in a heterogeneous sample. In this study, we intended to assess the accuracy of point-of-care capillary and venous/arterial samples to venous/arterial samples analyzed in the laboratory in critically sick patients who were either shocked or not shocked.

## Main text

### Material and methodology

This is a prospective case–control study of 268 critically ill patients hospitalized to the King Fahd Military Medical Complex in Dhahran, Saudi Arabia. Inclusion criteria comprised any adult, non-pregnant patients aged 18 or older who were hospitalized to the hospital with or without diabetes millitus and whose key decision makers provided informed consent. Exclusion criteria included hypovolemic shock due to significant active bleeding, bleeding disorders, the use of substances that could interfere with POC glucose meter technology (such as icodextrin-containing solutions, intravenous immunoglobulins, abatacept, and maltose), and lack of consent.

In this study, demographic characteristics such as age, gender, and anthropometric measures, as well as previous data such as chronic comorbidities, were acquired from a record review in addition to baseline laboratory findings. Patients’ initial vital signs, the Glasgow Coma Scale score with derived verbal scores for intubated patients, the necessity for a ventilator or dialysis, and the presence of acute severe arrhythmias comprised their baseline clinical features. In the laboratory, arterial blood gas analysis, serum creatinine, albumin, and a complete blood count were recorded. The laboratory data obtained closest in time to the blood glucose measurement were recorded.

Three blood samples were taken from the venous, arterial, and central blood vessels, as well as the capillaries, in order to collect data on the findings of POC and lab analysis of blood for glucose concentrations. We gathered and utilized data regarding the outcomes of POC and laboratory analyses for our comparison. The tests were performed by the same bedside nurse at the request of the treating physician.

All data were entered, manipulated, and analyzed using SPSS version 26. Frequency and percentage were utilized to characterize categorical variables, whereas mean and standard deviation were employed to characterize continuous variables. T-paired test was used to analyze the potential difference between glucose measurements obtained from POC and lab analysis of blood. All statements with P values less than or equal to 0.05 are deemed significant.

## Results

We were able to collect data on 268 patients admitted to King fahad military Medical Complex, Dhahran, Saudi Arabia, for this study. 26.1% of these patients with shock were male, whereas 18.3% were female. The average age is 63, 01 years. 20% of the included stunned patients were in the heart department, whereas only 2.5% were in the neurosurgery department. In addition, 27.7% of the patients with shock were diabetic, 25.3% required hemodialysis, and 59.3% were ventilated (Table 1).

**Table 1:** Baseline characteristics

	Shocked		Non-shocked	
Gender				
Male	70 (26.1%)		97 (36.2%)	
Female	49 (18.3%)		52 (19.4%)	
Total	119 (44.4%)		149 (55.6%)	
Age	Mean (SD)		63.01 (17.26)	
Specialty				
Cardiology	54 (20.1%)		27 (10.1%)	
Endocrine	2 (0.7%)		4 (1.5%)	
Neurosurgery	7 (2.6%)		16 (5.9%)	
Pulmonolgy	6 (2.2%)		15 (5.6%)	
Other	9 (3.4%)		21 (7.8%)	
D.M 2				
Yes	73 (27.2%)		59 (22.01%)	
No	46 (38.6%)		90 (33.5%)	
Hemodialysis				
Yes	68 (25.3%)		128 (47.7%)	
No	51 (19.1%)		21 (7.9%)	
Mechanical ventilation				
Yes	59 (59.3%)		50 (18.7%)	
No	60 (22.3%)		99 (36.9%)	
Urine output (ml/h)	115.6	381.07	110.2	321.4
Urine output in last 24 h (ml)	2195.28	1813.3	2064.9	1720.2
Weight (Kg)	74.9	17.8	77.3	18.1
Glasgow coma Scale	20.1	191.9	18.3	181.1
Temperature	36.82	2.91	36.91	3.1
Heart rate	91.2	22.3	88.3	21.2
Capillary refill	73.4	0.8	72.1	0.7
Serum creatin	131.7	125.7	133.1	122.1
Albumin	44.0	176.2	41.7	171.3
Hematocrit	29.1	20.9	28.2	20.3
Hemoglobin	9.2	1.7	9.4	1.8
WBC	13.7	6.1	12.4	5.9
PH	7.40	0.3	7.37	0.2
PO2	115.9	487.2	116.8	491.2
Lactate	1.82	2.31	1.72	2.2
SOFA score	9.41	4.71	8.9	4.3
APACHE II score	26.3	21.9	25.9	22.1

Moreover, 78.5% of hospitalized patients require insulin infusion. In addition, we discovered that 55.6% of the patients were not shocked, while 44.4% were. In evaluation of patient baseline characteristics, all relevant data were listed in Table 2. In the previous 24 h, the mean Urine Output for the shocked patients was 2195.28 ml compared to 2064.9 ml for the non-shocked patients. The average weight of patients in the shocked group was 74.9 kg compared to 77.3 kg in the non-shocked group. The mean temperature of hospitalized patients was 36.82 degrees Celsius, and their average heart rate was 91.2 beats per minute. The Sequential Organ Failure Assessment (SOFA) has an average score of 9.41. On the basis of laboratory and clinical data, the SOFA score forecasts ICU mortality. The score for the Acute Physiology and Chronic Health Evaluation (APACHE 2) was 26.3. APACHE 2 assesses ICU mortality based on a variety of laboratory data and patient symptoms, taking into consideration both acute and chronic diseases.

**Table 2:** The difference between POCT and Lab results considering glucose concentration

	POC analysis		Lab analysis		P-value
	Mean	Standard deviation	Mean	Standard deviation	
Capillary	9.22	3.54	–	–	
Venous	10.18	5.13	10.05	5.34	0.389
Arterial	9.66	3.15	11.41	10.31	0.029*
Central	9.18	3.87	9.54	3.84	0.208

In Table 2, we compared the venous/arterial glucose readings of critically sick patients who had POC analysis against lab analysis. Point of Care Testing (POCT) and lab results of both venous and central blood showed no significant difference (P = 0.389 and 0.208), although POCT showed slightly higher results with venous glucose concentrations of 10.18 and 10.05 (POCT and lab tests respectively) and 9.18 and 9.54 (POCT and lab tests respectively). The main difference between POCT and lab analysis of glucose concentrations was seen in arterial glucose concentrations, where lab analysis revealed significantly higher glucose concentrations (P = 0.029) with 11.41 against 9.66 in POCT analysis (Table 2).

In addition, we compared the findings of POCT and Lab analysis of glucose concentrations in shocked and non-shocked patients in Table 3. The results demonstrated that there is no substantial difference between the results of POCT and lab analysis between patients who were shocked and those who were not, with only a tiny discrepancy between POCT and lab analysis. The POCT revealed that the venous glucose concentration was greater in non-shocked patients and lower in the case of capillary analysis; however, laboratory analysis revealed that the venous glucose concentration was greater in shocked patients and somewhat lower in the case of central analysis.

**Table 3:** The difference between POCT and Lab analysis in shocked and non-shocked patients

Condition of the patients		POC venous	POC capillary	Lab venous	Lab central
No shock	Mean	10.3525	9.6739	9.9810	9.5455
	N	61	56	58	11
	Std. Deviation	4.71606	3.6638	4.39655	3.76520
Shock	Mean	9.6955	9.7320	10.1829	9.6521
	N	22	107	17	21
	Std. Deviation	6.23809	4.1341	7.92549	3.97787
P-value		0.610	0.532	0.846	0.996

## Discussion

Patients on a strict glycemic protocol and at increased risk of hypoglycemic episodes must have their glucose levels measured accurately. This is generally the case in this study’s population, as many of these patients are unable to interact with physicians or nurses, and their hypoglycemia symptoms are not readily available. POCT has a number of advantages over conventional blood glucose testing, including the availability of glucose values to the nurse within two minutes and immediate visibility in the hospital information system. In addition, POCT devices require a negligible volume of blood and the risk of blood spillage from the syringe or the device is minimal [20].

In order to implement a protocol for glucose regulation, it is necessary to measure blood glucose levels rapidly and accurately [21, 22]. The application of these protocols increases the nurse’s burden, hence it must be feasible [12, 13, 23]. This implies that not the most exact equipment, but one that is the most practical and provides reasonably accurate glucose analysis would be chosen for this process. In critically ill patients, however, hypoglycemia is critical, and its warning signals are missing; hence, these devices must also be highly reliable in the low range [14–19].

When we compared POCT results with lab analysis of glucose concentrations, we discovered that there is no difference between venous and central glucose, however arterial glucose concentrations differ significantly. In addition, we discovered that the POCT exaggerated the venous and central glucose concentrations, while underestimating the arterial glucose concentration. Petersen J et al. [24] and Boyd et al. [25] and Critchell et al. [19] similarly found that glucose meters overstated blood glucose levels in arterial, central, venous, and capillary samples relative to reference standard concentrations. In a different study conducted by Clarke et al. the authors found that the subcutaneous CGMS was accurate in the euglycemic range [26] and in a study conducted by Goldberg et al. they discovered that the POCT had a pearson correlation coefficient of 0.88 with 98.7% of patients falling within the clinically acceptable zones [27]. In a study conducted by Cook et al. the authors discovered that lab glucose values for blood from catheter in critically ill patients were significantly different from POC values for blood from catheter (P = 0.001) and fingerstick (P = 0.001) [14]. In addition, a second study revealed that the clinical agreement between POCT and laboratory analysis is greater in central blood analysis than arterial blood analysis, and in the case of hypoglycemia, only 26.3% of patients with capillary blood analysis demonstrated clinical agreement [16].

Despite the fact that hypoperfusion during shock is recognized to be a factor in the underestimating of glucose levels with capillary sampling [19, 21, 22], it is not observed to be a significant concern in this investigation. Considering glucose concentrations, for instance, there is no significant difference between shocked and non-shocked patients using POCT or laboratory analysis. This conclusion is comparable to the outcomes of earlier investigations [12, 13, 23].

We relied on critically sick patients in this study because we wanted to verify the reliability of POCT under certain situations, such as shock. Under high conditions of pH, temperature, electrolyte abnormalities, and hypoglycemia, there are consequently few data points from which to draw conclusions regarding the dependability of specific analyzers.

### Limitation of the study

The sample size was rather modest. This is a single-centre study. Blood was collected by various nurses. Bias induced by the design, manufacture, or use of a monitor.

In conclusion, Except for arterial blood glucose, the results of POCT and standard laboratory analysis of glucose concentrations in critically sick patients did not differ significantly in this investigation. Compared to laboratory blood analysis, the use of POCT is marginally accurate, with no difference between shocked and non-shocked patients.

## Data Availability

The data provided by the investigators was anonymized and processed. The following data, models, or code developed or used during the study are proprietary or private and may only be shared under certain conditions (e.g. anonymized data).

## Abbreviations

POC: Point of care
POCT: Point-of-care testing
ICU: Intensive care unit
KFMMC: King Fahd Military Medical Complex
